# Relationship between Oral Lichen Planus and Cardiovascular Disease of Atherosclerotic Origin: Systematic Review and Meta-Analysis

**DOI:** 10.3390/jcm13164630

**Published:** 2024-08-07

**Authors:** Beatriz Gonzalez Navarro, Sonia Egido Moreno, Carlos Omaña Cepeda, Albert Estrugo Devesa, Enric Jane Salas, Jose Lopez Lopez

**Affiliations:** 1 School of Dentistry, Oral Health and Masticatory System Group, (Bellvitge Biomedical Research Institute) IDIBELL, University of Barcelona, 08907 Barcelona, Spain; beatrizgonzalez@ub.edu (B.G.N.); albertestrugo@ub.edu (A.E.D.); enricjanesalas@ub.edu (E.J.S.); 2 School of Dentistry, University of Barcelona, 08907 Barcelona, Spain; soniaegido@ub.edu (S.E.M.); omanacepeda@ub.edu (C.O.C.); 3 Dental Hospital Barcelona University, Faculty of Medicine and Health Sciences (School of Dentistry), Campus Bellvitge, University of Barcelona, C/Feixa LLaga s/n, L’Hospitalet de Llobregat, 08907 Barcelona, Spain

**Keywords:** meta-analysis, oral lichen planus, OLP, cardiovascular disease, CVD, lipids

## Abstract

**Background/Objectives**: Oral lichen planus (OLP) is a chronic inflammatory autoimmune disease of the oral mucosa that affects between 0.5% and 2% of the general population. In the last decade, several studies have associated cardiovascular diseases (CVDs) with some inflammatory skin diseases such as oral lichen planus, demonstrating the presence of dyslipidemia in these pathologies. The objective of this work is to review whether patients with OLP show higher dyslipidemia and CRP levels compared to a healthy control population without OLP. **Methods**: Searches were carried out in Medline, Scopus, and Cochrane. The studies had to perform a histopathological diagnosis for OLP and the patients could not take any medication to treat this disorder. Non-lichenoid reactions were included. **Results**: After an initial search that provided us with 254 papers, this number was reduced to 10 articles after a detailed evaluation. All of them were case–control studies that compared the presence of analytical cardiovascular risk factors in patients affected by OLP and in healthy subjects. **Conclusions**: There is no scientific evidence of the possible association between OLP and CVDs. The only association we can prove is the one between OPL and CVD risk factors, especially those related to the lipid profile. More studies are needed in order to evaluate this relationship in patients diagnosed with CVDs.

## 1. Introduction

Oral lichen planus (OLP) is an autoimmune and chronic inflammatory mucocutaneous condition of the oral mucosa. Oral manifestations can be symptomatic or not, and they affect between 0.2% and 5% of the population, occurring more frequently in elderly women [1,2,3,4]. Geographically, the prevalence of OLP is higher in South America, Africa, and Europe. The prevalence actually increases significantly and progressively from the age of 40 [5,6]. It is the dermatological disease that most frequently presents via oral manifestations [7]. It has been shown that the immune system plays an important role in this disease, indicating that it is a pathology induced by a dysfunction of T cells [1,2,3], particularly CD8+ T cells [8].

The exclusive oral presentation of this disease occurs in one in every three patients, with the three most frequent locations being the buccal mucosa, the tongue, and gums (in the form of desquamative gingivitis) [9,10]. OLP tends to adopt different morphologies and experiences unpredictable periods of remission and exacerbation, persisting for years [11]. The different clinical forms in which it can manifest are mainly the reticular/non-erosive OLP, which includes the reticular, papular, plaque, and erythematous forms, which can either exhibit symptoms or remain asymptomatic, and the atrophic/erosive OLP, which includes ulcerative and/or bullous forms (not accepted by all authors), which is usually painful [10].

The diagnosis of OLP must be based on the recognition of the clinical manifestations, as well as performing a histopathological study in order to enable us to confirm the disease [12,13]. The histology of OLP is based on the following characteristics: hydropic degeneration of the basal layer due to liquefaction of the basal epithelial cells; intraepithelial and subepithelial band-like infiltrate of T lymphocytes and mononuclear cells, and the absence of epithelial dysplasia [14,15,16]. CD8 T cells are related to the liquefaction of basal cells, and these cells produce a complex network of cytokines and chemokines [17]. This inflammatory process that occurs just below the basal lamina (interphase stomatitis) and that may be present in other mucocutaneous diseases, is on the one hand critical to differentiate them from other common pathologies in the mouth, such as leukoplakia, and at the same time it is the inflammatory basis that determines the possible interrelation with chronic inflammation and systemic diseases [1].

Cardiovascular disease (CVD) is the leading cause of death in developed countries [18,19,20,21]. Under this category, we can find four entities: Coronary artery disease (CAD), sometimes referred to as coronary heart disease (CHD), results from decreased myocardial perfusion that causes angina, myocardial infarction (MI), and/or heart failure, representing half of the pathologies. The other types are cerebrovascular disease (CVD), including stroke and transient ischemic attack (TIA); peripheral artery disease (PAD), particularly arterial disease involving the limbs that may result in claudication; and aortic atherosclerosis, including thoracic and abdominal aneurysms. Although there are different etiologies, the most common cause is of atherosclerotic origin [22]. This etiology is multifactorial and inflammation-based, taking place after the accumulation of lipids and fibrous tissue in the arterial walls [22]. Different risk factors for this disease have been found, classified as conventional and unconventional factors [23]. Among the conventional factors, there is an increase in low-density lipoproteins (LDL-c), smoking, high blood pressure, and diabetes, among others. On the other hand, the unconventional risk factors include an increase in C-reactive protein (CRP) [24] and lipoprotein (a) [25], and the presence of oral pathology [26]; these have been identified more recently, and the evidence on their association with CVD is less extensive than the evidence that exists with conventional factors [23]. The epidermal cells in lichen planus have shown abnormalities in enzymatic activity, also in carbohydrate expression [27].

During the last few years, several studies have associated CVDs with some chronic inflammatory skin diseases such as systemic lupus erythematous [28], psoriasis [29], and lichen planus, showing lipid disorders in these patients [30]. The possible association between OLP and CVD could be related to systemic chronic inflammation [31]. Active psoriasis lesions reveal the infiltration of white blood cells, and many studies report higher levels of white blood cell activation products in the peripheral blood of these patients [32]. It has been found that the values of plasma inflammatory markers are increased in patients with OLP, which stimulates the procoagulant properties of endothelial cells, generating higher endothelial dysfunction, as well as stimulating the formation of foam cells and oxidative stress [33,34].

On the other hand, during the inflammation process, classic alterations occur in lipid metabolism [35], including an increase in triglycerides (TGs), very low-density lipoprotein (VLDL), total cholesterol (TC), and low-density lipoprotein (LDL-C), as well as a decrease in high-density lipoprotein (HDL-C), all due to the direct effect of the T cell responses [36]. If the inflammation becomes chronic, the changes in the lipid profile are maintained, such as in patients with OLP. In addition, raised levels of inflammatory markers such as CRP [37] and homocysteine are also shown [38]. Regarding acute coronary syndrome, it is most frequent in OLP patients with red lesions in comparison to patients with white lesions [39]. The etiopathogenic basis of this process could be an increase in oxidative stress linked to chronic inflammation [40].

It seems clear that patients with OLP experience a chronic inflammatory process and inflammation is present in the dyslipidemia–atherosclerosis equation. For this reason, the objective of this systematic review is focused on how to answer the PICO question of whether patients with OLP show higher dyslipidemia and CRP levels compared to a healthy control population without OLP.

## 2. Materials and Methods

This systematic review was conducted according to the guide of the Preferred Reporting Items of Systematic Reviews and Meta-analyses (PRISMA) statement [41]. A detailed protocol was prepared and registered in Prospero (ID: 571574).

### 2.1. Focused Question

Is there a relationship between OLP and CVD risk factors?

### 2.2. PICO Question

P: adult healthy patients; I: patients with OLP; C: patients without OLP; O: patients with LPO that have higher values of cardiovascular parameters (total cholesterol, CRP, LDL-C, HDL-C, TGC).

### 2.3. Eligibility Criteria

The inclusion criteria for the review were observational case–control or cohort studies conducted on humans over 18 years old. The OLP diagnoses had to be made with a biopsy and histopathological study. The evaluation of the cardiovascular risk factors was carried out through blood samples (total cholesterol, CRP, LDL-C, HDL-C, and TGC). Articles published in English where the patients were not under active OLP treatment (corticoid or palliative drugs) or were diagnosed with lichenoid reactions were also eligible. Finally, articles in which there was a self-reported history of a CVD or OLP diagnosis were discarded.

### 2.4. Search Strategy

A literature search was carried out without a time limit until the end of March 2024. The databases consulted were MEDLINE (pubmed), Scopus, and Cochrane Library. A partial gray literature search was also performed. The used keywords were as follows: “Oral Lichen Planus” AND “Cardiovascular disease”; “Oral Lichen Planus” AND “Inflammatory markers”; “Oral Lichen Planus” AND “Homocysteine”; “Oral Lichen Planus” AND “Lipids”; “Oral Lichen Planus” AND “oxidative stress”; “Oral Lichen Planus” AND “Dyslipidemia”. There was no time limit.

### 2.5. Study Selection

All articles were reviewed by two authors (BGN, SEM). After screening the titles, the articles in which the abstract met the inclusion criteria were selected. The full text of these studies was read by all authors to select the articles that met the inclusion and exclusion criteria. Disagreements during the selection process were resolved by consulting two other authors (JLL, EJS).

### 2.6. Data Extraction and Method of Analysis

The data were independently extracted by two authors (BGN, SEM), and in the case of disagreements, a third author (EJS) was consulted to obtain a consensus. The data referring to the following parameters were extracted: author(s), year of publication, country, type of study, number of patients with OLP and number of control patients, age, sex, and the evaluated analytical parameters.

### 2.7. Quality Assessment

The Newcastle–Ottawa scale (NOS) for assessing the quality of non-randomized studies in meta-analyses was implemented to evaluate the different sources of bias in the selected case–control studies; a “star system” was developed in which a study was judged in the following 3 domains: selection of case and controls, comparability of cases and controls, and ascertainment of exposure. The rating was as follows: Good quality: 3 or 4 stars in the selection domain, 1 or 2 stars in the comparability domain, and 2 or 3 stars in the outcome/exposure domain; fair quality: 2 stars in the selection domain and 1 or 2 stars in the comparability domain and 2 or 3 stars in the outcome/exposure domain; and poor quality: 0 or 1 star in the selection domain or 0 stars in the comparability domain or 0 or 1 stars in the outcome/exposure domain [42]. The evidence levels of the articles found will be cataloged according to the level of evidence and grade of recommendation of the Oxford Centre for Evidence-Based Medicine (CEBM) [43].

### 2.8. Statistical Analysis

The synthesis of the results was performed using a random-effects approach. Primary effect measures were the mean difference in cardiovascular biomarkers between patients with and without OLP. The statistical method for combining the results of individual studies was the inverse variance. Heterogeneity among studies was assessed using the I2 statistics and was considered statistically significant for a *p*-value < 0.1. A guide to interpreting the I^2^ statistic is provided in the Cochrane Handbook, where 0–40% is considered unimportant, 30–60% may represent moderate heterogeneity, 50–90% represents substantial heterogeneity, and 75–100% is considerable heterogeneity. If the data obtained allowed it, analysis was carried out by subgroups. Each outcome was combined and calculated using the Review Manager software (version 5.4). If any of the data obtained represent significant clinical relevance, it will be specifically stated in the results.

## 3. Results

### 3.1. Study Selection

**Figure 1:**
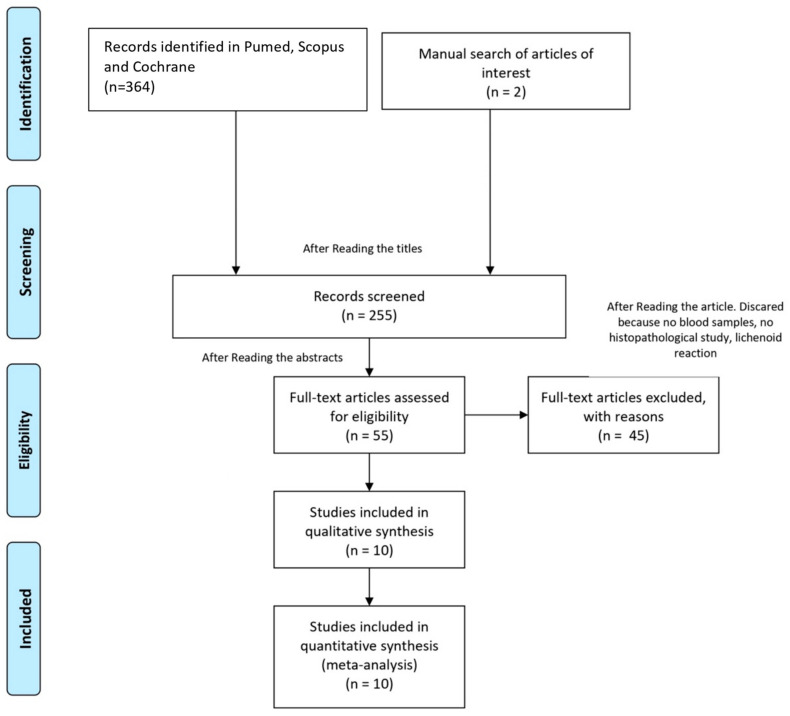
Flow chart.

Using the keywords, our search provided us with a total of 364 articles. After a detailed evaluation, we finally selected 55 articles. The gray literature did not provide additional articles. After, we discarded duplicate articles, reviews, and those that did not fit with the inclusion criteria (no blood samples, no histopathological study for LPO, lichenoid reaction). We included two more studies, extracted from manual searching because we considered them to be important for this review. Therefore, we included 10 studies (Figure 1). The included studies did not show conflicts of interest regarding their financing.

### 3.2. Studies’ Methods and Characteristics

All selected studies were case–control [30,31,35,36,37,44,45,46,47,48] (Table 1). The total number of patients across all studies was 1556, of which 858 had some type of lichen planus (LP), and 698 were part of a control group. Concerning the patients with LP, 634 had OLP, and the others had a cutaneous manifestation of LP (CLP) [30,31,35,36,37,44,45,46,47,48]. The sample was made up of 1008 women and 488 men, and one of the studies, which involved 60 patients, did not specify the sex of its population [35]. The mean age was 49.03 years, with a range of 40.12 to 62.41 years [30,31,36,37,44,45,46,47,48]. In a study by Aniyan et al. [35], the patient’s age was not specified. Three of these studies were carried out in a European population [27,40,42], while the rest of them presented samples with Asian individuals [30,35,36,37,44,45,47]. Only three studies took into account and divided the sample between patients with OLP in non-erosive/reticular OLP and erosive/atrophic OLP [44,45,46]. Table 2 summarizes the analytical parameters analyzed in the studies. We found that six of them analyzed CRP [30,31,36,37,44,45], eight analyzed LDL-C [30,31,35,36,45,46,47,48], eight analyzed TG [30,31,35,36,45,46,47,48], five analyzed TC [30,31,46,47,48], four analyzed glucose [31,36,45,46], four analyzed TC/HDL-C [30,31,46,48] and eight analyzed HDL-C [30,31,35,36,44,45,46,47]. Finally, three authors studied the index LDL/HDL [30,31,48].

### 3.3. Quality Assessment

The quality assessment showed differences between the included studies. All the studies showed between six and seven stars in the NOS; two or four in the selection of case–control domain, one in the comparability of the case–control domain and three in the ascertainment of the exposure domain [42]. Table 3 represents the quality assessment using NOS [42]. According to the NOS [42], six stars are considered to represent a low-quality study and seven stars represent a good-quality study. All in all, four studies presented as good quality [30,31,35,48] and the other six are considered low-quality studies [36,37,44,45,46,47]. The level of evidence and grade of recommendation were 3b/B. This review meets 22 of the 27 items of the PRISMA statement [41].

### 3.4. Synthesis of Results

**Figure 2:**
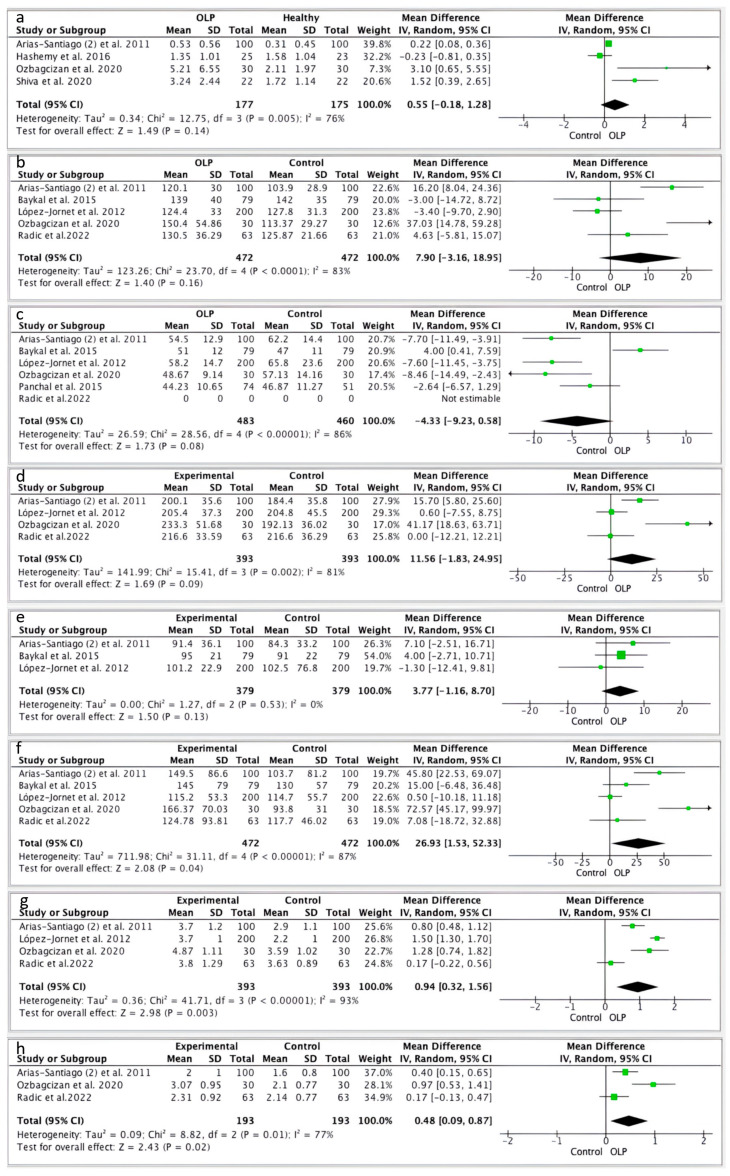
Forest plots of meta-analysis summarizing the results. (**a**) CRP in patients with and without OLP; (**b**) LDL in patients with and without OLP; (**c**) HDL in patients with and without OLP; (**d**) TC in patients with and without OLP; (**e**) glucose in patients with and without OLP; (**f**) TG in patients with and without OLP; (**g**) TC/HDL-c in patients with and without OLP; (**h**) LDL-c/HDL-c in patients with and without OLP. Each individual result has been presented in “green” and square shaped to better visualize its weight in the final result. And to differentiate it from the final result, presented as a black diamond, [30,31,36,37,44,45,46,48].

**Figure 3:**
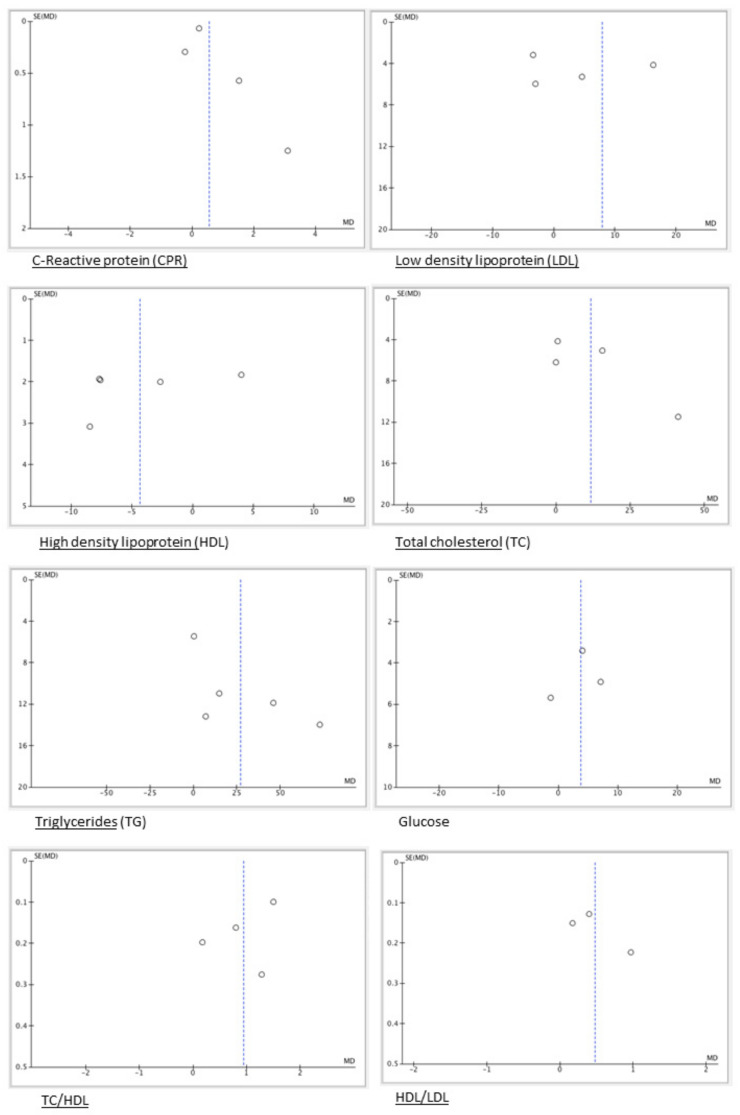
Funnel plots to assess risk of bias. The circles represent the jobs and their degree of dispersion with respect to the expected average, in dashed blue line.

In the pooled analyses, there were no statistically significant differences in CRP values between patients with OLP compared to those without OLP (mean difference: 0.55 95% CI −0.18, 1.28 *p* = 0.14) (Figure 2a). Results from the meta-analyses also showed that patients with OLP did not present statistically significant higher levels of LDL (mean difference: 7.9 95% CI −3.16, 18.95, *p* = 0.16) (Figure 2b), HDL (mean difference: 4.33 95% CI −9.2, 0.58, *p* = 0.08) (Figure 2c), TC (mean difference 11.56 95% CI −1.83, 24.95, *p* = 0.09) (Figure 2d), and glucose (mean difference: 3.77 95% CI −1.16, 8.70, *p* = 0.13) (Figure 2e); although these results were not statistically significant, the group OLP presented higher concentrations of the parameters than the control group. On the other hand, the result was statistically significant in TG values between OPL patients and healthy patients, with higher concentrations (mean difference: 2.93 95% CI 1.53, 52.33, *p* = 0.04) (Figure 2f). Finally, we found statistically significant differences in the formulas TC/HDL-c and LDL-c/HDL-c. Patients with OLP had higher levels of TC/HDL-c (mean difference 0.94 95% CI 0.32, 1.56, *p* = 0.003) (Figure 2g) and LDL-c/HDL-c (mean difference 0.48 95% CI 0.09, 0.87, *p* = 0.01) (Figure 2h). A substantial heterogeneity among the results was also detected, except in glucose levels (I^2^ = 0%). A funnel plot of every parameter was created to assess the publication bias (Figure 3).

## 4. Discussion

Based on our main objective of analyzing the relationship between OLP and CVD of atherosclerotic origin, the present systematic review demonstrates that there is no study that really evaluates the relationship between OLP and CVD, although it does show the existence of alterations in analytical parameters considered to be CVD risk factors (TC, HDL-C, LDL-C, TG, CRP, glucose, and TC). For this reason, we decided to review the studies that analyzed these parameters in blood samples taken from patients with OLP. These samples were used to check if their levels were in the ranges considered to be normal or altered. In this way, it is possible to demonstrate the association between these two diseases.

The majority of the reviewed studies concluded that there is a relationship (not causality) between OLP and CVD risk factors, despite it not always being statistically significant [30,31,35,36,37,44,45,46,47,48]. What most studies do agree on is that chronic inflammation plays a crucial role in the development of CVD risk factors, and OLP is clearly an inflammatory disease. Thus, chronic inflammation is the common pattern between both entities [1].

The chronic inflammation present in immune-mediated diseases leads to discrepancies in lipid metabolism [49]. The inflammatory cascade activation induces a decrease in HDL-C and phospholipids. These lipid disturbances could stimulate compensatory changes, such as the synthesis and accumulation of phospholipid-rich VLDL, resulting in hypertriglyceridemia [49].

Chronic inflammation, oxidative stress, and lipid disorders cause an increase in the prevalence of CVD [21,22]. According to the review by Godoy-Gijón E [49], patients affected by OLP have one to three times more risk of suffering from CVD. Therefore, inflammation is considered to be an important contributor to atherothrombosis. For this reason, the measurement in the serum of inflammatory markers could be important to diagnose this disease in patients affected by OLP [46].

Several studies state that higher values of TG and low levels of HDL-C were associated with the transition from atheroma to atherothrombosis and therefore, the control of these two CVD risk factors is essential in patients with subclinical disease [31]. Furthermore, the classic lipid changes associated with the metabolic syndrome (increased TG and decreased HDL-C) may become a CVD risk factor [35].

Generally, we can identify two principal subtypes of OLP, namely, erosive OLP and reticular OLP. Several studies discuss that erosive OLP could be a higher risk factor than reticular OLP to develop CVD [44,45,46]. This conclusion is based on the fact that erosive OLP is an ulcerative, atrophic oral disorder with more tissue damage; therefore, it is much more symptomatic, causing intense discomfort to the patient. These characteristics lead to a higher presence of pro-inflammatory cytokines, which involves a higher inflammatory state. Therefore, differences between OLP subtypes demonstrate the different degrees of inflammation and their importance in the possible induction of CVD [44,45,46]. We could not carry out a meta-analysis investigating the differences between erosive and reticular OLP because we were not able to find the homogeneous parameters in the reviewed literature, as only one of these articles reported CRP [44], one other study did not divide into subgroups [45], and another article was not comparable because, despite dividing into subgroups, it was a single study [46].

It is noteworthy that the different clinical presentations of LOP are easily distinguishable and its recognition could help to better approach and follow up with patients with cardiovascular disease, at least in patients at risk.

The articles in which patients took some type of medication were discarded because the drugs used to treat dyslipidemia have an important impact on CVD risk factors. In addition, some of these drugs are associated with a rash similar to lichen planus (which can be considered a lichenoid reaction). On the other hand, many drugs used to treat OLP like retinoic acid, methotrexate, or systemic corticosteroids are also associated with the development of dyslipidemia, and in general worsen cardiovascular risk parameters [49]. Therefore, they could be considered to present a risk of bias for this review.

This study has some limitations. The interpretation of the results of this meta-analysis must be very carefully performed, since the articles are very heterogeneous. The patients in the evaluated studies were not diagnosed with CVD; they had OLP and they showed alterations in some parameters that are considered to be CVD risk factors. Therefore, they will have a higher risk in the future of developing this disease. In the majority of the analyzed studies, another important limitation was the adjustment for other potential confounders, which not all authors made. This can lead to a biased result from which to draw conclusions about the association of OLP and CVD. So, the results must be interpreted with caution.

## 5. Conclusions

There is no scientific evidence of the possible association between OLP and CVD. The only association we can prove is the one between OLP and CVD risk factors.

Chronic inflammation plays a crucial role in the development of CVD risk factors. For this reason, we have to take into account these inflammatory markers. In OLP, the cardiovascular and metabolic risk factors are frequently altered due to chronic inflammation.

Despite the limitations of the studies and the limited bibliography that has been evaluated, it is suggested that OLP can play an important role as a new risk factor for concomitant dyslipidemia and atherosclerosis, despite there not being any clear relationship.

Finally, it is necessary to carry out more studies, with larger samples of patients affected by OLP, which evaluate all CVD risk factors in order to obtain statistically significant conclusions. In addition, in order to be able to relate these diseases, there is a need for studies with patients diagnosed with CVD. With these criteria, we can check if the association between these diseases exists and the greater presence or absence of OLP in patients affected by CVD.

## Figures and Tables

**Table 1:** Main results of the studies.

Author/Year	Country	Type of Study	N Patients	Mean Age	Sex
Li et al., 2023 [47]	China	C.C.	100 OLP 75 other mucosal disorders 100 healthy	47.06	181 women 94 men
Radic et al., 2022 [48]	Croatia	C.C.	63 OLP—63 healthy	62.41	103 women 23 men
Shiva et al., 2020 [37]	Iran	C.C.	22 OLP—22 healthy	46.2	25 women 19 men
Ozbagcivan et al., 2020 [30]	Turkey	C.C.	30 CLP 30 OLP 30 CLP with OLP (COLP) 30 healthy	48.01	77 women 43 men
Aniyan et al., 2018 [35]	India	C.C.	30 OLP—30 healthy	NS	NS
Hashemy et al., 2016 [44]	Iran	C.C.	25 OLP—23 healthy 13 OLP reticular 12 OLP erosive	45.09	33 women 15 men
Panchal et al., 2015 [36]	India	C.C.	74 LP—51 healthy 74 CLP 10 OLP	40.12	67 women 58 men
Baykal et al., 2015 [45]	Turkey	C.C.	79 LP—79 healthy 55 OLP 17 OLP erosive 38 OLP reticular	47	100 women 58 men
López-Jornet et al., 2012 [46]	Spain	C.C.	200 OLP—200 healthy 155 OLP reticular 45 OLP erosive	57.61	322 women 78 men
Arias Santiago et al., 2011 [31]	Spain	C.C.	100 LP—100 healthy 69 OLP	47.85	100 women 100 men

C.C.: Case–control study; OLP: oral lichen planus; CLP: cutaneous lichen planus; COLP; cutaneous and oral lichen planus; LP: lichen planus; NS: not specified.

**Table 2:** Parameters studied by the different studies analyzed.

Author/Year	CRP	c-LDL	TG	TC	Glucose	TC/c-HDL	c-HDL	LDL/HDL
Li et al., 2023 [47]		X	X	X			X	
Radic et al., 2022 [48]		X	X	X		X	X	X
Shiva et al., 2020 [37]	X							
Ozbagcivan et al., 2020 [30]	X	X	X	X		X	X	X
Aniyan et al., 2018 [35]		X	X				X	
Hashemy et al., 2016 [44]	X							
Panchal et al., 2015 [36]	X	X	X		X		X	
Baykal et al., 2015 [45]	X	X	X		X		X	
López-Jornet et al., 2012 [46]		X	X	X	X	X	X	
Arias Santiago et al., 2011 [31]	X	X	X	X	X	X	X	X

CRP: C-reactive protein; c-LDL: low-density lipoprotein; TG: triglycerides; TC: total cholesterol; c-HDL: high-density lipoprotein.

**Table 3:** Quality assessment of the analyzed studies according to Newcastle–Ottawa scale [42] and level of evidence and grade of recommendation of CEBM [43].

Author/Year	Selection of Cases and Controls	Comparability of Cases and Controls	Ascertainment of Exposure	Conclusion	Evidence and Recommendation of CEBM
Li et al., 2023 [47]	★★	★	★★★	Low quality	3b/B
Radic et al., 2022 [48]	★★★	★	★★★	Good quality	3b/B
Shiva et al., 2020 [37]	★★	★	★★★	Low quality	3b/B
Ozbagcivan et al., 2020 [30]	★★★	★	★★★	Good quality	3b/B
Aniyan et al., 2018 [35]	★★★	★	★★★	Good quality	3b/B
Hashemy et al., 2016 [44]	★★	★	★★★	Low quality	3b/B
Panchal et al., 2015 [36]	★★	★	★★★	Low quality	3b/B
Baykal et al., 2015 [45]	★★	★	★★★	Low quality	3b/B
López-Jornet et al., 2012 [46]	★★	★	★★★	Low quality	3b/B
Arias Santiago et al., 2011 [31]	★★★	★	★★★	Good quality	3b/B

All the studies showed between six and seven stars in the NOS; two or four in the selection of case–control domain, one in the comparability of the case–control domain and three in the ascertainment of the exposure domain.
